# Ancient technology and punctuated change: Detecting the emergence of the Edomite Kingdom in the Southern Levant

**DOI:** 10.1371/journal.pone.0221967

**Published:** 2019-09-18

**Authors:** Erez Ben-Yosef, Brady Liss, Omri A. Yagel, Ofir Tirosh, Mohammad Najjar, Thomas E. Levy

**Affiliations:** 1 Department of Archaeology and Ancient Near Eastern Cultures, Tel Aviv University, Tel Aviv-Yafo, Israel; 2 Department of Anthropology, University of California, San Diego, La Jolla, California, United States of America; 3 Levantine and Cyber-Archaeology Lab, University of California, San Diego, La Jolla, California, United States of America; 4 Institute of Earth Sciences, Hebrew University of Jerusalem, Jerusalem, Israel; University at Buffalo - The State University of New York, UNITED STATES

## Abstract

While the punctuated equilibrium model has been employed in paleontological and archaeological research, it has rarely been applied for technological and social evolution in the Holocene. Using metallurgical technologies from the Wadi Arabah (Jordan/Israel) as a case study, we demonstrate a gradual technological development (13^th^-10^th^ c. BCE) followed by a human agency-triggered punctuated “leap” (late-10^th^ c. BCE) simultaneously across the entire region (an area of ~2000 km^2^). Here, we present an unparalleled, diachronic archaeometallurgical dataset focusing on elemental analysis of dozens of well-dated slag samples. Based on the results, we suggest punctuated equilibrium provides an innovative theoretical model for exploring ancient technological changes in relation to larger sociopolitical conditions—in the case at hand the emergence of biblical Edom–, exemplifying its potential for more general cross-cultural applications.

## Introduction

Archaeology is uniquely suited through its deep-time perspective to holistically and diachronically examine ancient human, social, and technological evolutions. Changes in technology provide unique opportunities for quantitatively measuring the refashioning of material culture through time to monitor underlying social change. From the perspective of metallurgical technologies in the Southern Levant, for example, archaeological research has identified the inception and sustained growth of copper metallurgy since circa 5000–4000 BCE [1–3]. However, while moments of radical metallurgical transformation, such as novel casting technologies and the innovation of bronze, have also been identified [1], there has been little investigation of small-scale changes in technological practices; although requiring considerable research efforts, the identification and modeling of such technological evolutions have broad significance in archaeological research, as technology can function as a proxy for otherwise indiscernible social processes [4]. Recent archaeological research in the Wadi Arabah [5, 6], one of the largest copper ore resource zones in the Levant, allowed for investigating in unprecedented detail a continuous record of technological development that spans a period of significant social changes in the transition between the Late Bronze Age (LB) to the Iron Age (IA).

**Fig 1:**
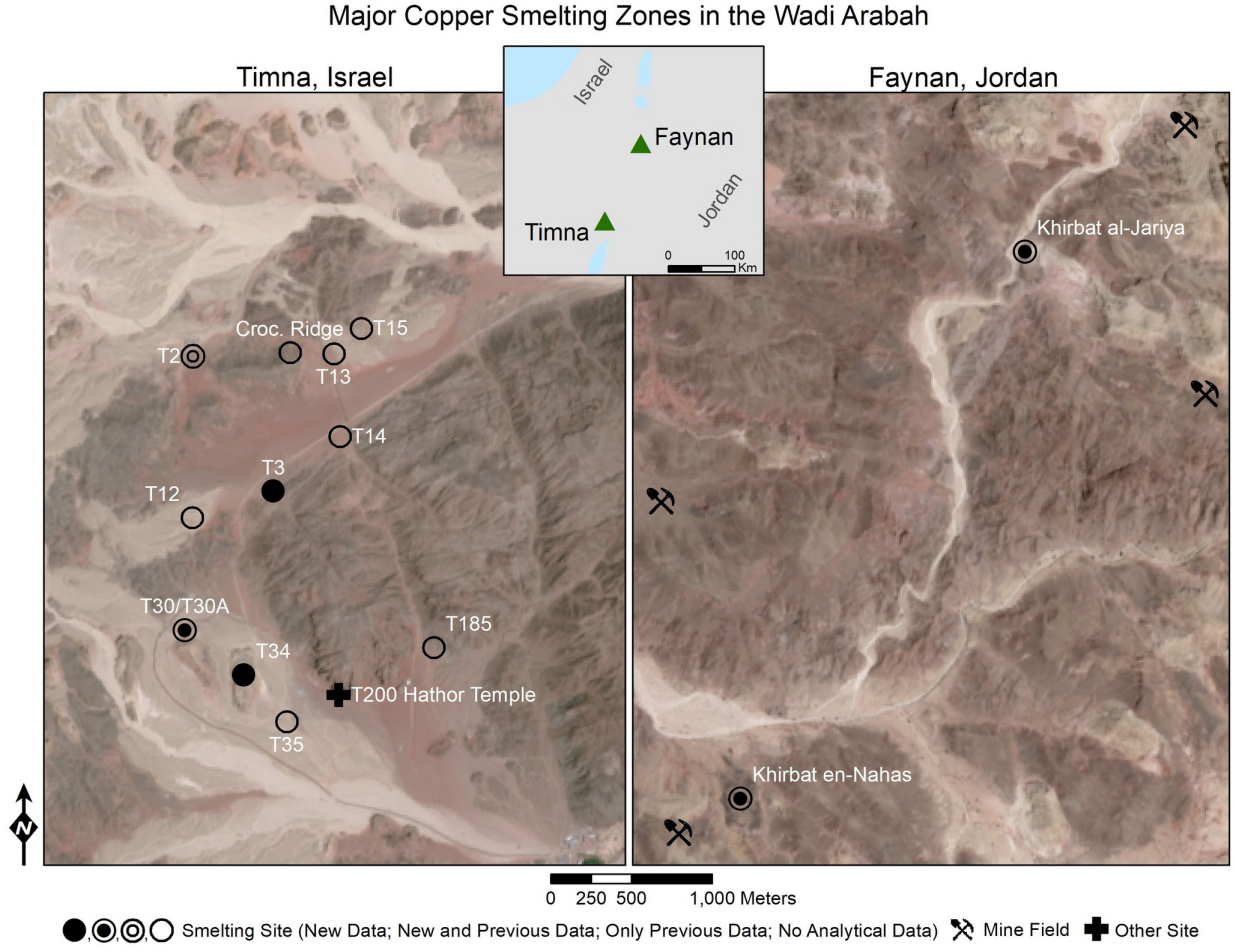
Map of main copper producing regions in the Wadi Arabah (Israel/Jordan). Location of copper smelting sites discussed in text (detailed maps in S1 and S2 Figs; analytical results of previous studies are compiled in S1 Dataset). Map produced using ArcGIS software by ESRI. Sentinel-2 (ESA) image courtesy of the U.S. Geological Survey (public domain).

Almost unharmed by modern exploitation, the industrial landscapes of Faynan and Timna within the Wadi Arabah (Fig 1, S1 and S2 Figs) function as exceptional “field laboratories” for examining developments in ancient metallurgical technologies [7, 8]. While previous research has demonstrated that the IA witnessed the most intense industrial activity in the history of the region—surpassing the later eastern Roman Empire operations, until now, it was impossible to measure technological change at the sub-century level to precisely map the peak in production and associate changes with social and cultural processes. During the IA, both regions were populated by large copper smelting centers supported by networks of smaller, ephemeral mining camps [5, 6]. These smelting sites remain identifiable on the surface by significant accumulations of metallurgical slag (>100,000/10,000 tons of slag in the Faynan and Timna respectively) [7, 8]. Slag was typically deposited in mounds mixed with other metallurgical debris; these constitute a unique, quasi-continuous record of the smelting activities (S3 Fig) [7, 8] that can typically be dated by radiocarbon measurements of charcoal (fuel remains) [9, 10] and archaeomagnetic investigation of the slag material itself [10, 11]. To explore these archaeological features, the Edom Lowlands Regional Archaeology Project (ELRAP—directed by T.E.L. and M.N. [5]) and the Central Timna Valley Project (CTV—directed by E.B.-Y. [6], http://archaeology.tau.ac.il/ben-yosef/CTV/) systematically excavated slag mounds in Faynan and Timna respectively. Securely dated slag samples from these excavations were analyzed as part of the current study, resulting in a robust archaeometallurgical dataset. In the following we demonstrate how the application of evolutionary theory helps extract social insights out of the new data and discuss the implications on our understanding of the genesis and structure of the Edomite Kingdom.

## Punctuated equilibrium and technological change

Proposed as an alternative to phyletic gradualism, the Darwinian process entailing new species evolving through gradual and steady transformations, punctuated equilibrium suggests biological evolution is characterized by rare, rapid, and episodic events of speciation (cladogenesis) [12, 13]. Between these speciation events, biological organisms and communities are believed to exist in stability or stasis, essentially unchanging [12]. This reinterpretation of the evolutionary process provided an improved explanation for the discontinuous fossil record which lacks the long sequences of intermediate forms between origin and ancestral species expected by phyletic gradualism [12]. Paleontologists continue to find punctuated equilibrium a useful model for identifying and explaining rapid speciation in the fossil record (e.g., [14], often complementing phyletic gradualism based on the recognition of stasis as a meaningful and predominant pattern within the history of species [15]. As with the fossil record, this evolutionary approach can assist in interpreting change or “evolution” within the “deep-time” archaeological record, contributing to a macro history understanding of parts of the world. In following, evolutionary perspectives based in biology and paleontology have significantly influenced the history and growth of archaeological theory [16, 17]. Traditionally, this theoretical borrowing was archaeologically applied to cultural phenomena in place of biological species, using evolutionary models to interpret/understand developments in material culture and social complexity [16].

Punctuated equilibrium in archaeology has been predominantly applied to cases from the Paleolithic period. Kolodny et al. [18] review a wide range of studies going back to the 1940s, that illustrate how gradually accumulated changes in prehistoric human stone tool assemblages can culminate in empirically punctuated “events” of invention/innovation or loss. However, as these studies deal with deep-time prehistory and processes of change, it is difficult to identify the role of human agency. Human agents driving punctuated change were identified by Schiffer [19], who—as part of his study of 19^th^ century electromagnetic technology—refers to technological change as invention “cascades” that produce new technologies, each cascade being a punctuated change that can be linked with specific inventors (human agents). O’Brian and Bentley [20] review a wide range of studies on the tempo of technological and social evolution in relation to Schiffer’s Behavioral Archaeology use of cascade models to explain the evolution of “complex technological systems”. Regarding ancient metallurgical technologies, evolutionary theory was only rarely applied, such as in the work of Charlton et al. [21, 22] concerning iron production in Wales. In the case at hand we apply evolutionary theory—and in particular punctuated equilibrium—in order to highlight, better characterize and make sense of detectable changes in copper production technologies, and in particular a “leap” in copper smelting efficiency that occurred during a very short period of time within a longer sequence of gradual changes. By considering the historical context, high precision radiometric dating and recent epigraphic finds, we identify a possible mode of change, i.e. the punctuation event that interrupted the preceding quasi-stasis.

## Investigating technological change in the Wadi Arabah

For this study, 154 slag samples collected from stratigraphically controlled contexts in Faynan and Timna were pulverized for homogenization and analyzed with a pXRF (n = 109), ICP-OES (n = 10) and ICP-MS (n = 35). The conceptual framework for investigating technological change in the Wadi Arabah was to diachronically and synchronically map slag chemical compositions in a period of seemingly continuous production in the region. The Cu contents of slag can function as a proxy for the efficiency of the smelting technology, especially in comparative observations (less Cu reflects improved efficiency, assuming no change in ore quality), while other elements can reveal additional technological information (e.g., flux usage) [7, 8].

## Results

Our results fall within the range of previously analyzed samples from LB and IA smelting sites of the Wadi Arabah (n = 72, S1 Dataset). The latter, lacking stratigraphic information and poorly dated, can be used only for a broad characterization of the period as a whole. Our new data, coupled with a tight control over well-dated stratigraphic contexts, enable investigation of technological change in a high resolution of time and space. A list of all radiocarbon dates used in this study, including ages modeled by Bayesian statistics where stratigraphic observations allowed, is provided in S2 Dataset.

**Fig 2:**
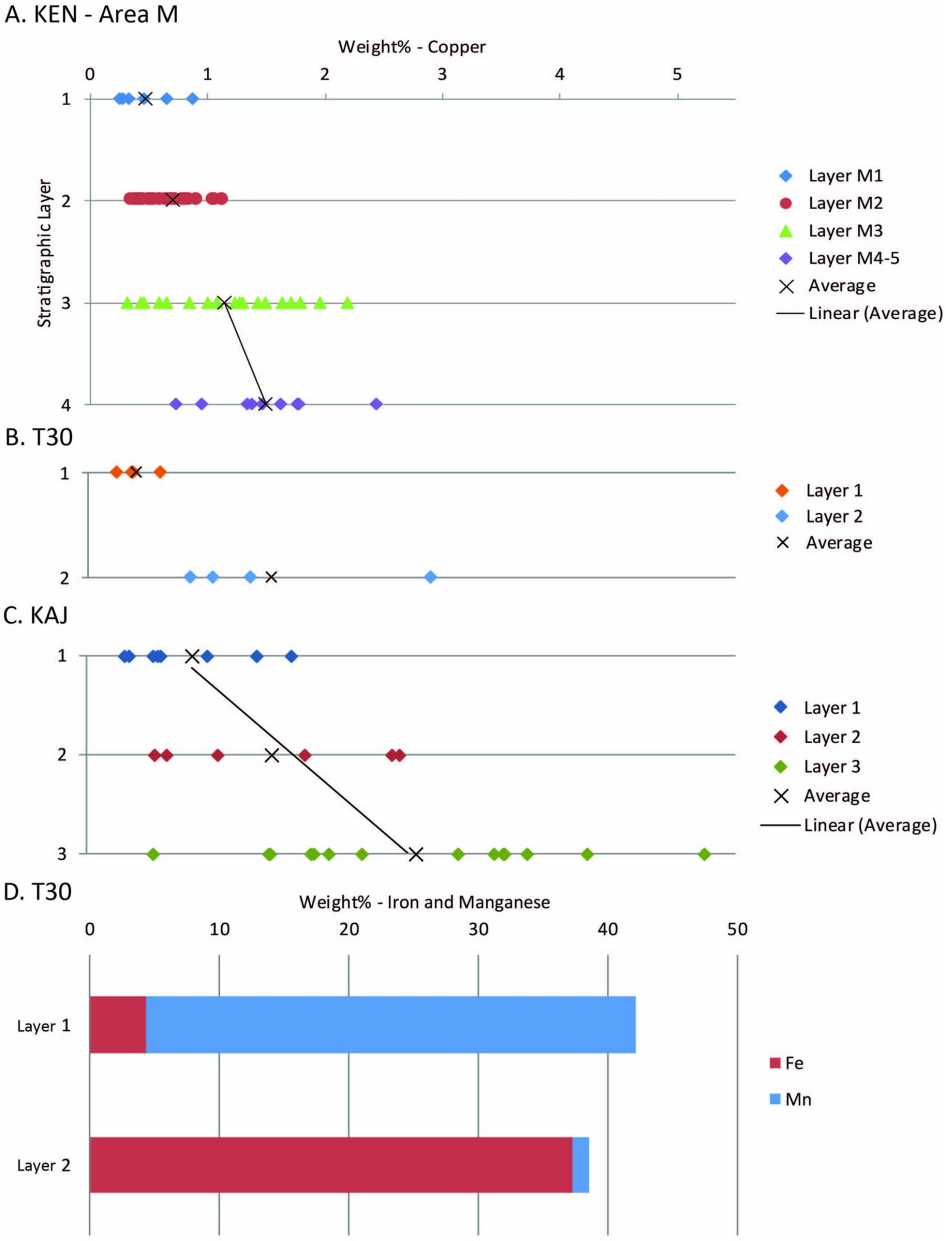
Elemental composition of slag samples by stratigraphic context at selected sites (data in S3 Dataset). A-C) Cu content of samples from Khirbat en-Nahas (KEN) Area M (A), Timna Site 30 [T30] (B), and Khirbat al-Jariya (KAJ) (C). Note the “leap” in efficiency and standardization between KEN M3 and M2, and T30 L2 and L1 (not represented at KAJ as the site was abandoned at the time). D) Mn and Fe content of samples from T30. The abrupt change between L2 (n = 3) and L1 (n = 4) reflects a deliberate replacement of the fluxing material (in Timna) that corresponds to the “leap” in efficiency noted in the Cu content.

**Fig 3:**
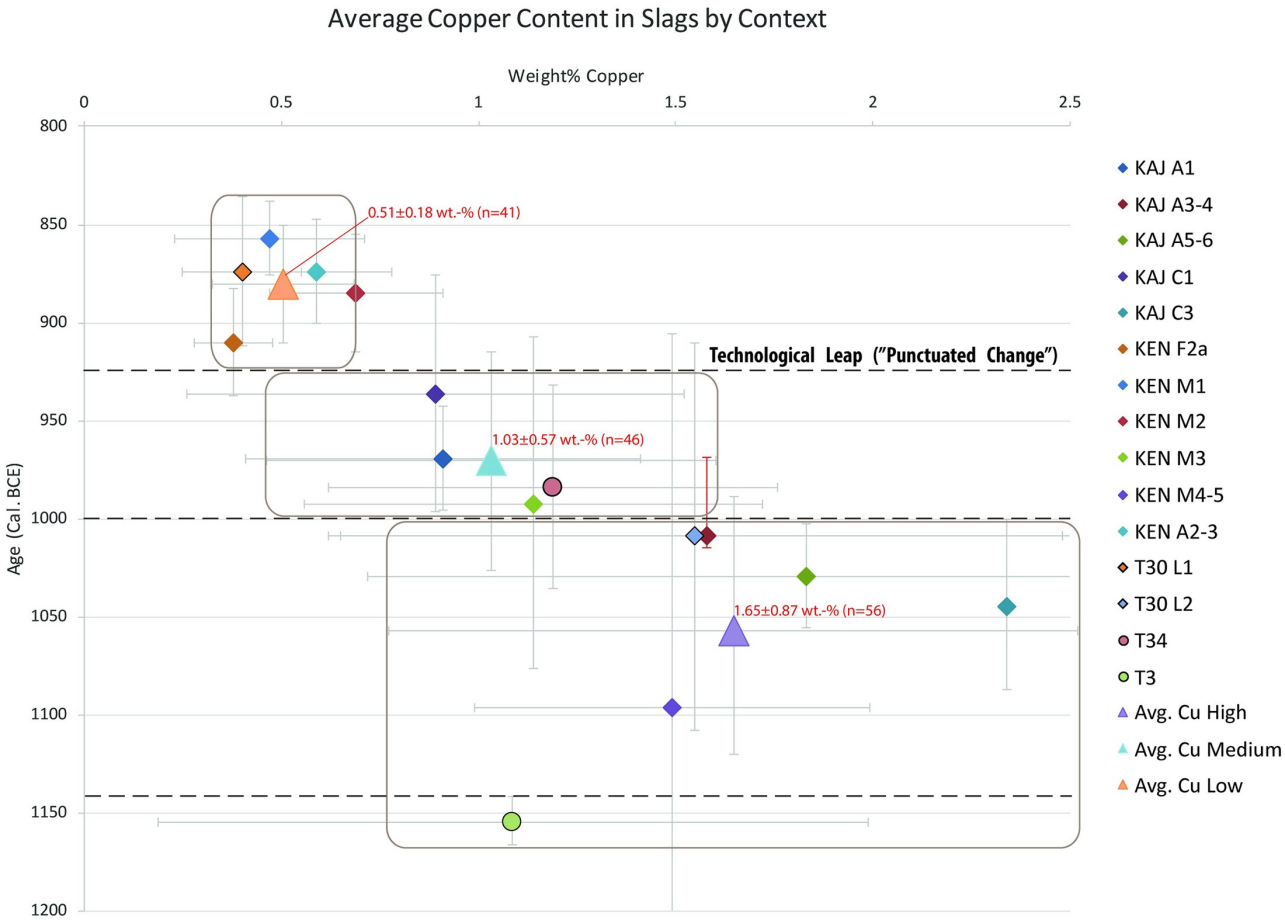
Average Cu content in slag samples by context plotted against associated radiocarbon dates (S2 and S3 Datasets; points from Timna are outlined in black, the rest are from Faynan). Points represented by a diamond are based on XRF data whereas circles are ICP-OES/MS data. The error bar of the x-axis represents the standard deviation, and the error bar of the y-axis is the maximum age range allowed by the available radiocarbon dates at 1σ (modeled by stratigraphy where applicable). Red error bars indicate a point that does not mark the median of the radiocarbon range, but is still within 68.2% probability. There are three distinct groups (marked by rounded rectangles) that demonstrate a gradual development in technology punctuated by a technological “leap”. This leap is represented here by a substantial reduction in average Cu content (a 55.5% change) and a significant decrease in the standard deviation, which is three times tighter for the post-leap average; note that the extent of the rounded rectangles along the x-axis represents the standard deviation. The dashed lines at 1140 and 925 BCE represent historical events (the Egyptian withdrawal from Timna and the military campaign of Pharaoh Sheshonq I, respectively), while the dashed line at 1000 BCE represents a time of general changes in the organization of production in the Wadi Arabah (see text and Fig 5).

Based on Cu content in slag, we were able to identify and contextualize a gradual improvement in technology within the LB-IA sequence that was followed by a “leap” that occurred sometime in the second half of the 10^th^ c. BCE (S3 Dataset, Figs 2 and 3); the correlation between Cu contents in slag and stratigraphic contexts is striking (Fig 2A–2C). This trend is evident, for example, in the deep sounding excavated in the slag mound of Khirbat en-Nahas (KEN), Area M (S3 Fig). The average Cu content in slag decreases gradually according to stratigraphy (Fig 2A, from 1.49±0.50 wt.-% [M4-5] to 01.14±0.58 wt.-% [M3]), with a rapid drop in the transition to the youngest layers (0.69± 0.22 wt.-% [M2] and 0.47±0.24 wt.-% [M1]). There is also a correlation between stratigraphy and the tightness of distribution of Cu contents, represented by the standard deviations [SD] of the measurements, suggesting a better control on the smelting technology through time. This pattern is definitely the case in KEN-Area M, where the transition between the “pre-leap” (ca. 1300–925 BCE) technologies (represented by M4-5 and M3, SD = 0.50 and 0.58 respectively) and “post-leap” (ca. 925–830 BCE) technology (represented by M2 and M1, SD = 0.22 and 0.24 respectively) was accompanied by a tightening of the standard deviation by a factor of two. That said, these observed trends are even more pronounced when all contemporaneous contexts across the Wadi Arabah are considered together (Fig 3).

A comparison between data from Faynan and Timna reveals another striking correlation, as the two regions, separated by ~105 km, demonstrate the exact same trend of technological changes (Figs 2 and 3; note for example the results from the contemporaneous sites of Timna 30 [T30] L2 and Khirbat al-Jariya [KAJ] A3-4 [1.55±0.93 wt.-%, 1.58±0.93 wt.-%, respectively], and T30 L1 and KEN M1 [0.40±0.15 wt.-%, 0.47±0.24 wt.-%, respectively]). In Timna, the elemental analysis revealed an abrupt change in the use of flux from Fe oxides to the more effective Mn oxides that accompanied the “leap” in efficiency (Fig 2D). In Faynan, where manganese oxides are associated with the main source of copper ore and more readily available, fluxing was done using these minerals throughout the period [8].

When copper content averages in slag samples are plotted by context (Site/Area/Layer), distinct clusters can be distinguished (Fig 3; see more on the chronological boundaries below). The large sample number enables further, statistical conclusions: (1) the low copper-content group discussed above (T30-L1, KEN-M1, KEN-M2, KEN-F2A, and KEN-A2-3) are not only tight at the context level, but also in the inter-context level (average of all samples from low copper-content contexts is 0.51±0.18 wt.-%); this means that in addition to having more control over smelting processes in the level of each individual workshop, there was a standard, and well-established technology across sites in the entire Wadi Arabah. (2) KEN-M3, KAJ-A1, KAJ-C1, and Timna Site 34 [T34] represent a cluster, with an average content of copper in slag of 1.03±0.57 wt.-%; also here there is an excellent agreement across contexts of similar dates across the Wadi Arabah, with technological tradition that lasted about a century around the turn of the 1^st^ millennium BCE. (3) The highest copper-content group (1.65±0.87 wt.-%), which is also the most scattered one, spans the transition between the LB and IA (ca. 1140 BCE [23]). Key observation here is that the transition is not reflected in the technology (note in particular the results of Timna Site 3 [T3], which—based on finds at the site—is considered to represent LB technology [23]).

**Fig 4:**
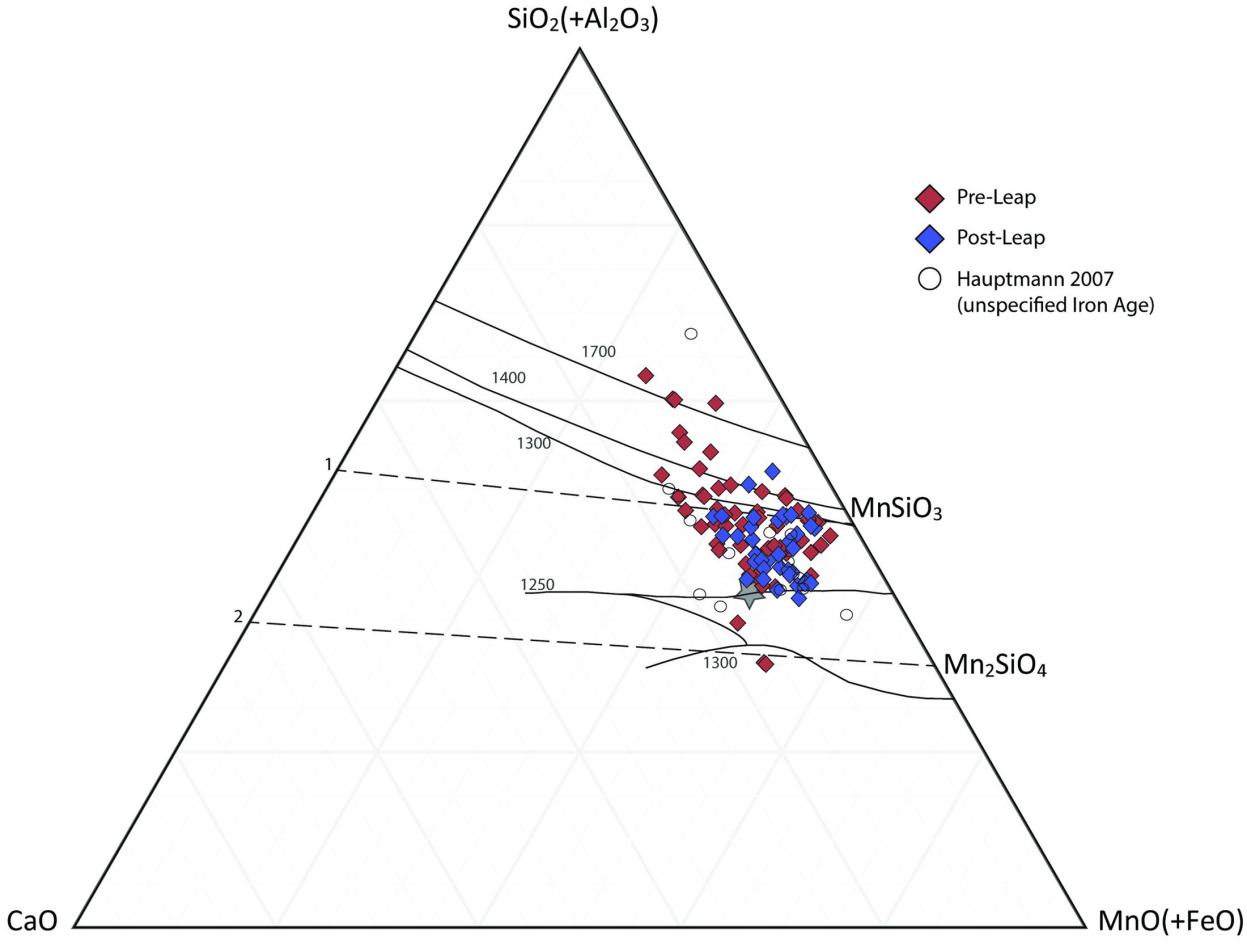
Results of Iron Age Mn-rich slag from the current study (all contexts in Faynan and T30 L-1 in Timna, S3 Dataset) and Hauptmann 2007 [8] (S1 Dataset) displayed in the system SiO2-CaO-MnO (after Glasser 1962 [24]). Primary phase boundaries are shown as solid black lines (values of nearby isotherms are also indicated), and the ternary liquidus minimum is marked by a grey star (~1200°C); also indicated are the lines of metasilicates (1) and orthosilicates (2). Most of the samples fall within and around the low melting temperature zone (the few data points plotted in the “forbidden” quartz-rich liquidous zone are likely derived from slag containing inclusions of unreacted quartz). The technological leap of the late 10th century BCE is visible here by the tightening of the scatter around the area that represents the more efficient smelting process (lower temperatures, lower viscosity). That said, all three datasets overlap, indicating that there was no major change in the main components of produced slag throughout the period. This understanding of the slag composition more generally supports using the Cu content in slag samples as a measure of smelting efficiency (cf. Figs 2 and 3). It is also worth noting the striking agreement between Hauptmann’s dataset (produced by Atomic Absorption techniques) and ours (the plotted data here were produced by pXRF).

Finally, the technological leap is evident also when considering the bulk chemical composition of the analyzed slag (Fig 4). Although the major chemical components were basically the same throughout the period (an observation that strengthens the use of Cu content as a proxy for technological change), the improvement in standardization and the overall efficiency of the smelting process is readily apparent.

## Discussion

### Archaeological and historical context

**Fig 5:**
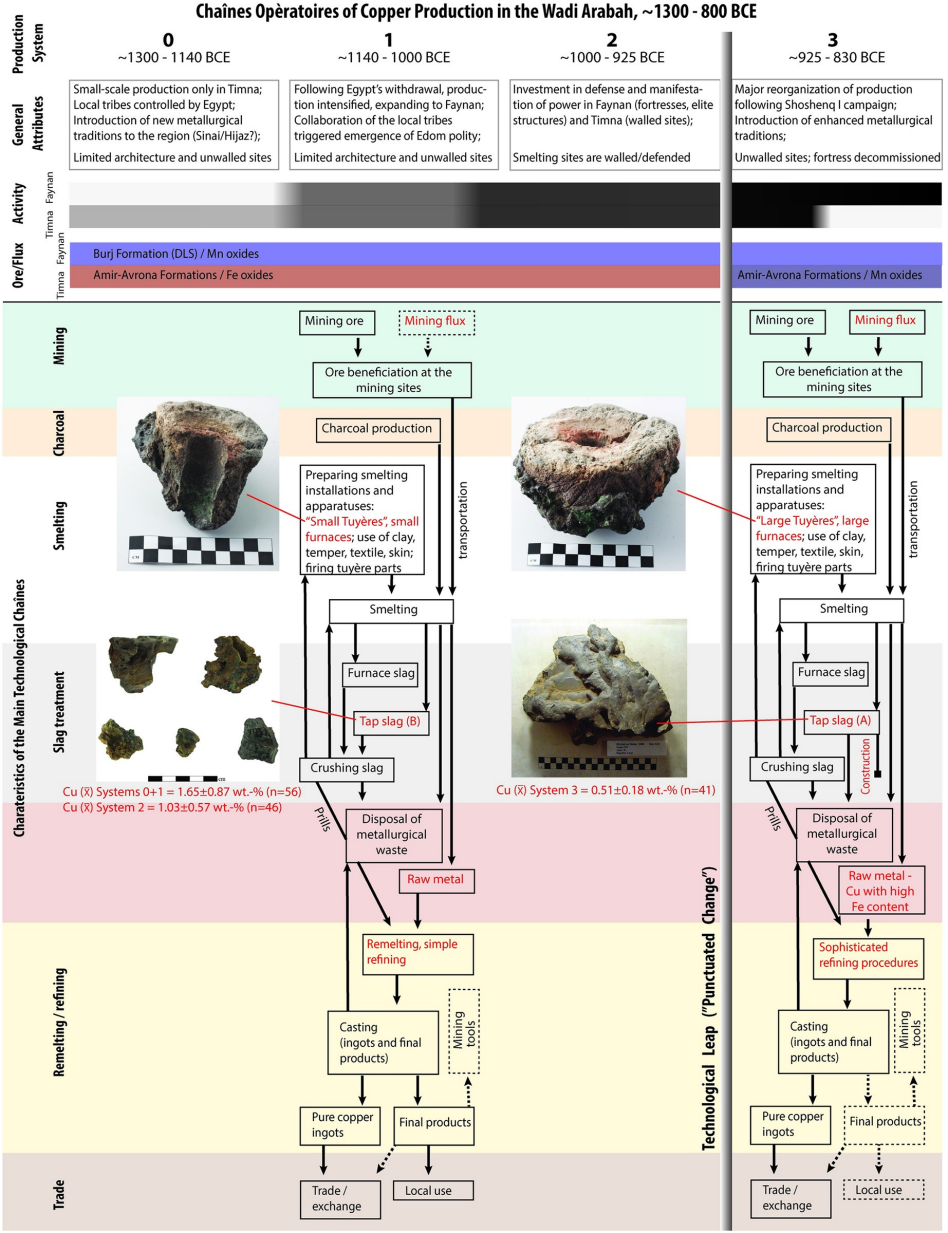
Main features of the organization of production and detailed chaîne opératoires for the copper industry in the Wadi Arabah during the Late Bronze and early Iron Ages (dashed arrows and boxes indicate optional components with weak or no evidence). Although attempts for improvements were probably inherent to the technological practice throughout the period, except from the “punctuated change” they were limited and contained within a given system of feedbacks (both positive and negative). These stabilizing feedbacks are associated with the various components of each chaîne opératoire, from constraints imposed by co-dependency of technological habits (for example, specific preparation of a smelting mixture dictated a specific smelt) to those of the existing trade organization and market demands. While some degree of gradual improvement in the period preceding the technological leap (Systems 0–2) is detectable in the slag chemistry (cf., Fig 3), it is only the latter that left macro evidence for changes (marked here in red and illustrated by tuyères and slag types [more on the macro evidence and the archaeometallurgical material culture associated with this leap, see [37]; on slag types, see [10]]). Radiocarbon dating and other archaeological observations indicate no significant break in production during the technological transformation; the change was based on the existing system that was striving for improvements and was “given the opportunity” to break loose from previous constraints by an external intervention (Shoshenq I’s campaign). There is no reason to assume that the change was orchestrated by any others than the Edomites themselves, as a positive response to disruption; the latter provided a “window” for incorporating “foreign” innovations (which included improvements in other technologies as well, most notably the introduction of camels to facilitate transportation, see [33]). It should also be noted that the techno-social evolution presented here is bounded by centuries of hiatus in the preceding and following periods: it started earlier in the south as a result of Egyptian initiative during the days of Seti I [38] and ended with the rekindling of Cypriot monopoly on Eastern Mediterranean copper trade, probably with the support of the Aramaeans [25]. The industry in the south probably ended somewhat earlier because of rapid deforestation caused by the practice of the intense, post-leap industry in the extreme arid region of Timna (where in contrast to Faynan there is no immediate sources of hydrophilic flora, see [39]).

The technological record studied here represents half-a-millennium of continuous copper production in the Wadi Arabah (~1300–800 BCE), which was preceded and followed by centuries of hiatus [5, 10, 25] (Fig 5). As such, this record provides a unique window into evolutionary processes of a given society; this society, identified with the Edomites of the Old Testament (Hebrew Bible) and non-biblical sources (Assyrian and Egyptian) [9, 26, 27], was based on the local tribes of the region that practiced a (semi-)nomadic way of life throughout the period [26]. The beginning of copper production took place in Timna under the control of the Egyptian New Kingdom [7]. The technologies, which were disparate from the wind-based ones used in the preceding Early Bronze production in the region (3^rd^ millennium BCE), were introduced from the outside, possibly based on developments in the Sinai Peninsula or the Hijaz [5, 28]. The subsequent, local technological developments were based on these technologies, in the center of which there were furnaces operated by sack-bellows [5].

The Egyptian-controlled production phase in Timna has no equivalent in Faynan and should probably be understood as an extension of the Egyptian activities in the Sinai Peninsula [28]. The absence of a New Kingdom Egyptian activity in the northern Wadi Arabah may be due to distance-parity and the inability of this core civilization to project their power in this area [29]. The degree of the Egyptian ‘hands-on’ involvement in the production activities is a matter of active discussion; however, it is evident that the labor force was predominantly based on the local tribes, working under the Egyptian hegemonic power that at the time was exercised over the core regions of the Southern Levant (Canaan). Following the collapse of the Egyptian civilization at the end of the LB [30] and the consequent Egyptian withdrawal from Timna (ca. 1140 BCE [23]), metal production spread throughout the Wadi Arabah at an industrial scale [5, 10]. From this time until the end of the large-scale industry around ca. 800 BCE, two major transformations are evident in the archaeological record (Fig 5): (1) An introduction of fortifications around the turn of the 1^st^ millennium BCE (e.g., the large fortress at KEN), which also entailed abandonment of unprotected sites (e.g., Timna Site 15) and the establishment of new ones in naturally fortified locations (e.g., “Slaves’ Hill” [T34] [31, 32]). The need for defense was manifested simultaneously in Timna and Faynan, and probably reflects changes in the ultra-regional geopolitical situation at the time [31]. Based on macro observations, these changes are not reflected in the technological practice, and the basic ‘toolkit’ remained the same [5]. (2) A substantial reorganization of the industry at the second half of the 10^th^ century BCE [5], which included efforts for centralization (abandonment of most sites except one in Timna and two in Faynan), decommissioning of defensive features (e.g., the transformation of KEN fortress into metallurgical workshops), the introduction of camels as draft animal [33], and major changes of organization on an intra-site level (e.g., establishment of a building in an area previously used for dumping metallurgical waste [KEN Area M]). These changes were accompanied by the introduction of a new technological ‘toolkit’, which consisted of larger furnaces and tuyères, and produced a visually-different type of slag [5]. It has been suggested that this major transformation should be associated with the campaign of Sheshonq I to the Southern Levant [5, 10].

Pharaoh Sheshonq I, one of the few Egyptian pharaohs identified by name in the Hebrew Bible (there “Shishak”), was the founder of the 22^nd^ Dynasty in Egypt. Following his ascension, he dedicated efforts to reunifying Egypt and incursions abroad [34]. His military campaigns included an invasion into Judah and Israel “5 years after the death of King Solomon” (1 Kings 14:25, 2 Chronicles 12:1–12) in response to hostile incidents on Egypt’s eastern border. This event is commonly dated to around 925 BCE, although difficulties with the biblical background for this date and insecurities regarding the exact years of Sheshonq I’s reign gave rise to slightly different (usually earlier) suggestions (e.g., [35]). Based on the appearance of names from the Negev region in the description of this event in Egypt (topographical list at the Temple of Amun in Karnak) it has been suggested that one of the destinations of Shoshenq I’s campaign was the Wadi Arabah and its copper industry [36]. This hypothesis was strengthened recently by the accidental discovery of a rare scarab bearing the throne name of Sheshonq I in Faynan [5].

The coincidence of Shoshenq I’s campaign to the region and the major reorganization of the copper industry mentioned above, together with the assumption that Egypt’s interest was to secure its supply of Arabah copper [28], suggests that Egypt had a certain role in bringing about these observable changes in the copper production industry. The new data presented here, coupled with punctuated evolution theory, help shed light on that role vis-à-vis internal processes within the local society.

### Technological evolution and the Edomite society

Treating technological developments from an evolutionary perspective assumes that a positive feedback for improvements in efficiency (= less efforts spent per unit of outcome) exists in a given techno-social system. This assumption should be treated with caution in the study of technological trajectories in human societies [40], as discrete cases within the *longue durée* of a given region can easily deviate from a unilinear development model [41]. However, for a continuous record of the same system such as the one studied here, this assumption seems valid, and an evolutionary model can be suggested.

Prior to the present study only two copper smelting technologies were identified in the LB/IA archaeological record of the Wadi Arabah, the one represented by the “Small Tuyères” (~1300–925 BCE) that was followed by the one represented by the “Large Tuyères” (~925–800 BCE) [5, 7, 8] (Fig 5). A ‘stasis’ in technological development was assumed for the duration of each of the technologies, and the advantages of the newer technology was discussed only in relation to increased production intensity based on the amount of slag. The new data (Fig 3) provide quantifiable observations on the efficiency of the two technologies; they also reveal hitherto indiscernible diachronic technological changes and help characterizing the macroscopically visible technological transformation.

The analytical data (Fig 3) demonstrate that the earlier technology was not entirely static, and that during the ca. 400 years of use it went through changes, indicating that efforts to improve its efficiency were deliberately—and probably constantly—invested. The changes were gradual and thus are best discernable in the general trend; however, within this trend the data suggest that significant technological changes were associated with the reorganization of the industry around the turn of the 1^st^ millennium BCE. The appearance of fortifications and redistribution of smelting sites at this time coincides with a tighter control over the smelting protocol (cf., SD of averages of Cu-High and Cu-Low in Fig 3), and an overall more efficient technology. Prior to these changes the standardization of production across the Wadi Arabah was rather poor, reflecting a different practice for each smelting site. Furthermore, the seamless transition from the Egyptian-controlled industry to the local (Edomite) one around ca. 1140 BCE supports the hypothesis that the tribes of the region were responsible for operating the industry even when it was orchestrated by the Egyptians; it reveals that under the Egyptian auspices the local tribes had a high degree of independence, a situation that constituted a fertile ground for them to emulate Egyptian political practices that would later serve as the basis for the consolidation of their own polity (cf., [6, 26].

The new technological observations also shed light on the latter process, the formation of the tribal confederation of the Edomite Kingdom. While the biblical narrative describes an early, pre-10^th^ century BCE kingdom (“…the kings who reigned in Edom before any Israelite king reigned” [Genesis 36:31]), the archaeological record has been subjected to conflicting interpretations, even after the publication of the new chronology that clearly demonstrates the flourishing of the region during the 12^th^– 11^th^ centuries BCE [5, 10, 42]. Here, the striking synchronous agreement between the technology in Timna and Faynan, evident as early as the 11^th^ century BCE (note in particular T30-L2 and KAJ-A3-4 in Fig 3), suggests that an overarching political body existed in the region already at this time. Further centralization of this political body is evident in the changes observed towards 1000 BCE mentioned above.

The ca. 400-years of gradual technological improvements, which from an evolutionary perspective can be regarded as a quasi-stasis, was “punctuated” by a technological “leap” in the second half of the 10^th^ century BCE. The major, macro-scale changes described above are reflected in the analytical results, which indicate a much more efficient technology that was practiced with unprecedented control over the smelting protocol (Fig 3); after the “leap”, the technology reached an “equilibrium”, continuing for more than a century with no discernable changes. The historical context makes human agency a key factor in explaining this “punctuation event”: after generations of internal efforts to better the technology—with limited success—the techno-social system was receptive of extraneous influences that facilitated the same cause. Thus, changes imposed or triggered by the Egyptian intervention at the time of Shoshenq I’s campaign resulted in unparalleled flourishing of the industry, rather than a catastrophic clash. This is evident not only in the industrial landscapes but also in the nearby regions that thrived as a result of trade with Egypt [43] and even further destinations such as Greece [25, 44].

The triggering of the technological leap by extraneous intervention is best explained when considering the characterization of periods of stasis in punctuated equilibrium theory. Such periods are stable a result of feedback systems that prevent change, and the punctuation is a result of destabilization of these systems. The feedback systems in the case at hand (Fig 5) are related to the complexity of the technological operation—which was based on multitude of co-dependent components—and the embeddedness of the industry in an existing trade system(s); only when these were destabilized by a military campaign (that probably also reflects changes in markets and trade) an opportunity emerged for the Edomites to adopt innovations and make technological changes that no doubt had—in turn—profound effects on their society. The case study of the Wadi Arabah demonstrates how technological developments can be interpreted through the lens of punctuated evolutionary theory; this not only helps in extracting social insights on a given society (in our case, Edom), but is a useful tool for comparative studies; its potential depends on its application to other detailed, high resolution records of technological change across time and cultures, world-wide.

## Materials and methods

Slag samples for the present study were obtained by excavation methods detailed in [5] and [6], and their dating is based on previously published radiocarbon results (S2 Dataset). As slag is typically a heterogeneous material, all samples were pulverized into a very fine homogenized powder (particles of several tens of microns) that provides a reliable average of the bulk chemistry of a sample. The chemical analysis was based principally on the use of a portable X-ray fluorescence (pXRF) instrument (n = 109). Inductively coupled plasma optical emission spectrometry (ICP-OES) and inductively coupled plasma mass spectrometry (ICP-MS) were also used for additional 10 and 35 samples respectively; the agreement between the results strengthens their accuracy.

1. **pXRF:** Instrument used: **Bruker TRACeR III-V+** (Levantine Archaeology Laboratory, University of California San Diego). In order to gain quantitative results from the produced XRF spectra, we calibrated the instrument using samples of slag with known elemental values (all of the samples were Mn-rich slag in order to account for this unique quality of slags in Faynan). Samples were pulverized using an agate mortar and pestle and a SPEX alumina ceramic grinder, in the Petrographic Laboratory of Scripps Institution of Oceanography. Powdered samples were collected in specialized cups with transparent thin-film bases (four microns thick). Focusing on heavier elements, copper and above on the periodic table, the Bruker pXRF was set to 40Kv, 15μA, no vacuum, and the green filter for timed assays of 300 seconds. Cu values discussed in this paper are much greater than the estimated detection limit for Cu using these settings.
2. **ICP-OES, ICP-MS:** All samples were pulverized using Retsch Jaw Crusher BB200 and Retsch Vibratory Disc Mill RS200, both equipped with tungsten components (Archaeometallurgical Laboratory, Institute of Archaeology of Tel Aviv University). The homogenized powders were stored in sealed bags and transferred to the Geochemistry Laboratory of the Institute of Earth Science, the Hebrew University of Jerusalem (headed by Y. Erel). From each sample, 0.1g was put into separate beakers and dissolved using multiple acids (HNO_3_ [69% concentration], HF [49% con.], HClO_4_ [35% con.]). After vaporizing the acids, all beakers were filled with 0.5ml of nitric acid and 49.5ml of distilled water. The chemical composition of 10 samples was then analyzed by Perkin Elmer Optima 3000 Inductively Coupled Plasma Optical Emission Spectroscopy (ICP-OES) and due to machine availability the rest 36 samples were analyzed using Agilent (HP) 7500cx ICP- Mass Spectroscopy (MS).

Provenience of archaeological samples used in the current study:

1. Samples stored in the University of California San Diego Levantine Archaeology Laboratory (available for study, metadata for Khirbat en-Nahas is available as a digital collection at- https://library.ucsd.edu/dc/collection/bb41653353):
  - Samples excavated under license number 2006/74 of the Department of Antiquities of Jordan: L170-1, L174-1, L193-1, L180-1, L180-1-C, L192-1, L816-1, L842-1, L859-1, L860-1, L860-1-C, L893-1, 10482, 10483, 10250, 10484, 10485, 10487, 10478, 10479, 10481, 10486, 10488, 10489, 10256, 10475, 10476, 10477, 10490, 10492, 10493, L602-1, L606-1, L615-1, L620-2, L620-1, L622-1, 10259, 10260, 10262, MAR0904, 10267, 10270, 10474, 10468, 10460, 10461, 10462, 10480, L629-1, L647-1, L659-1, L660-1, 10463, 10465, 10467, 10471, L666-2, L666-1, 10277, 10280, 10282, 10283, L667-1, L670-1, L670-2, L671-1, 10464, 10466, L674-1 // 3275, 3262, 3263, 3278, 3261, 3283, 3276, 3287, 3266, 3267, 3281, 3282, 3280, 3279, 3277, 3269, 3274, 3272, 3271
  - Samples excavated under license number 2014/60 of the Department of Antiquities of Jordan: B10314_1, B10314_2, B10338_1, B10338_2, B10233, B10234, B10235, B10236, B10237, B10238, B10502, B001
2. Samples stored in the Tel Aviv University, Laboratory of Archaeometallurgy (available for study):
  - Samples excavated under license number G-38/2009 of the Department of Antiquities of Israel: L809-1, S2-510, S2-51, S2-52, L902-1, L903-1, L907-1, L808-1
  - Samples excavated under license number G-3/2013 of the Department of Antiquities of Israel: oyT3;02;P, oyT3;03;C, oyT3;04.1;C/P, oyT3;04.2;C/P, oyT3;04.3;C/P, oyT3;04.4;C/P, oyT3;05.1;C/P, oyT3;05.2;C/P, oyT3;05.3;C/P, oyT3;05.4;C/P, oyT3;06.1;C/P, oyT3;06.2;C/P, oyT3;06.3;C/P, oyT3;06.4;C/P, oyT3;07.1;C/P, oyT3;07.2;C/P, oyT3;07.3;C/P, oyT3;07.4;C/P, oyT3;08;C/P, oyT3;09;C/P, oyT3;10;C/P, oyT3;11;C/P, oyT3;12;C/P, oyT3;13;C/P, oyT3-14-C/P, oyT3-15-C/P, oyT3-16-C/P, oyT3-17-C/P // oyT34;10.1;C/P, oyT34;11/1;C/P, oyT34;11/2;C/P, oyT34;12.1;C/p, oyT34;12/2;C/P, oyT34;13.1;C/P, oyT34;13/2;C/P, oyT34;14.1;C/P, oyT34;14/2;C/P, oyT34;15/1;C/P, oyT34;15/2;C/P, oyT34;16/1;C/P, oyT34;16/2;C/P, oyT34;17/1;C/P, oyT34;17/2;C/P, oyT34;18/1;C/P, oyT34;18/2;C/P

## Supporting information

S1 Fig Detailed map of Iron Age copper production and related sites in Faynan, Jordan. The white square represents the extent of the map provided in Fig 1. WF = Wadi Fidan, RHI = Rujm Hamra Ifdan, RAM = Ras al-Miyah. Map produced using ArcGIS software by ESRI. Sentinel-2 (ESA) image courtesy of the U.S. Geological Survey (public domain).
(TIF)

S2 Fig Detailed map of the Late Bronze and Iron Age copper production and related sites in Timna, Israel. The white square represents the extent of the map provided in Fig 1. The site numbers are based on the work of the Arabah Expedition. Map produced using ArcGIS software by ESRI. Sentinel-2 (ESA) image courtesy of the U.S. Geological Survey (public domain).
(TIF)

S3 Fig Students excavating metallurgical debris in a deep sounding of a slag mound in Faynan, Jordan (Khirbat en-Nahas [KEN], Area M). The rapidly-accumulating slag constitutes a quasi-continuous record of technological change (in this case, spanning 300 years) that can be tightly tied to chronology based on radiocarbon dating of charcoal (remains of fuel) and archaeomagnetic investigation of the slag material itself. The technological “leap” detected by the current study is represented here by the reorganization of the area during the second half of the 10^th^ century BCE: the construction of the building on the left, and the leveling-up of the earlier slag mound represented as a ‘disruption’ in the section at the level of the building’s foundation.
(TIF)

S1 Dataset A compilation of all previously published analytical data of Late Bronze and Iron Age slag samples from the Wadi Arabah. (XLSX)

S2 Dataset Radiocarbon dates (original, calibrated and modeled) from contexts discussed in the text and their sources. (XLSX)

S3 Dataset Analytical data from current study of Late Bronze and Iron Age slag samples from the Wadi Arabah by archaeological context (cf., S2 Dataset for dating). (XLSX)
