# Supplementary figures and images for: Ancient technology and punctuated change: Detecting the emergence of the Edomite Kingdom in the Southern Levant
Source: PLoS One. 2019 Sep 18;14(9):e0221967. doi: 10.1371/journal.pone.0221967 (PMC6750566; doi:10.1371/journal.pone.0221967)

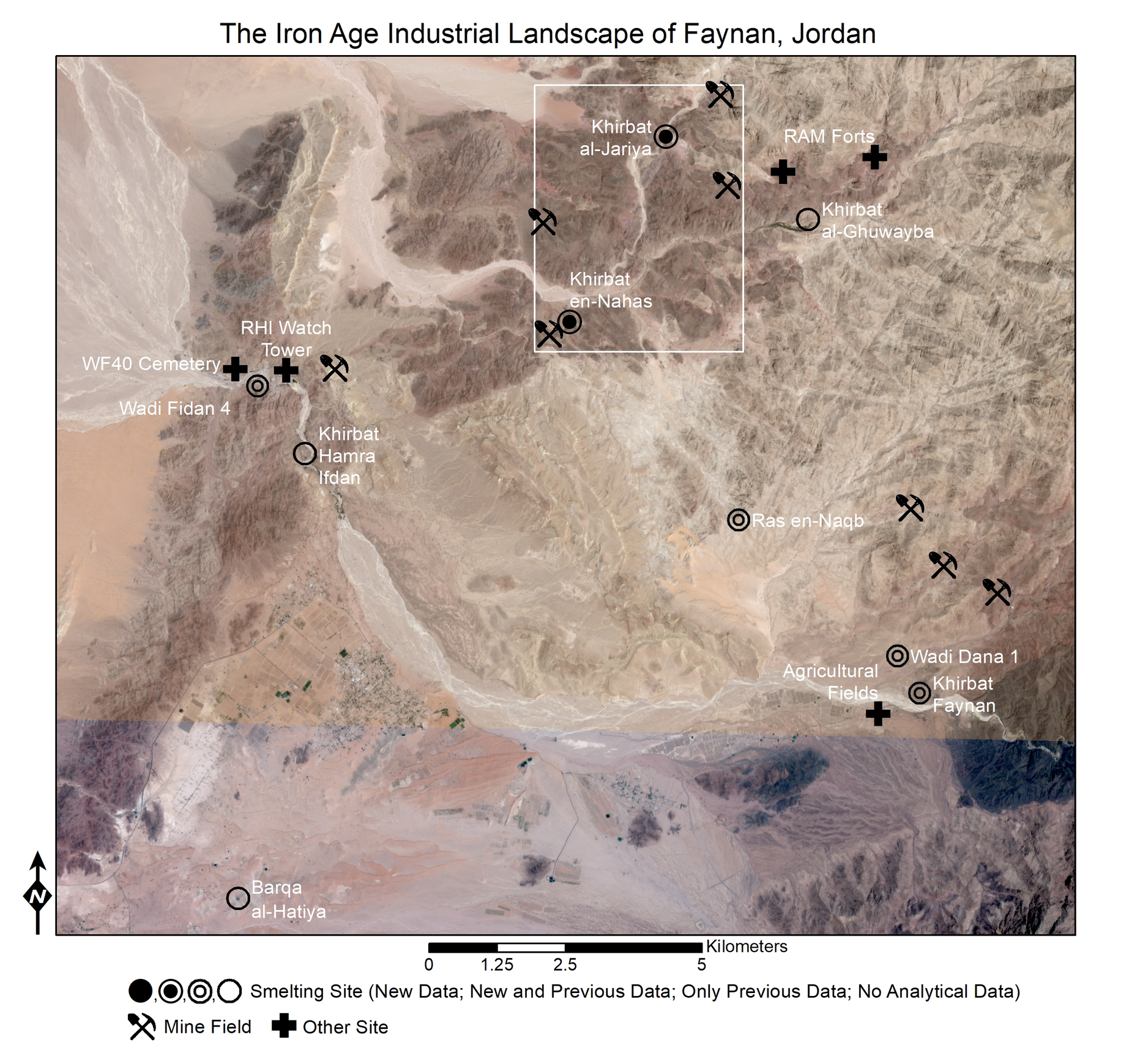

Supplement: S1 Fig — The white square represents the extent of the map provided in Fig 1. WF = Wadi Fidan, RHI = Rujm Hamra Ifdan, RAM = Ras al-Miyah. Map produced using ArcGIS software by ESRI. Sentinel-2 (ESA) image courtesy of the U.S. Geological Survey (public domain). (TIF) [file pone.0221967.s001.tif]

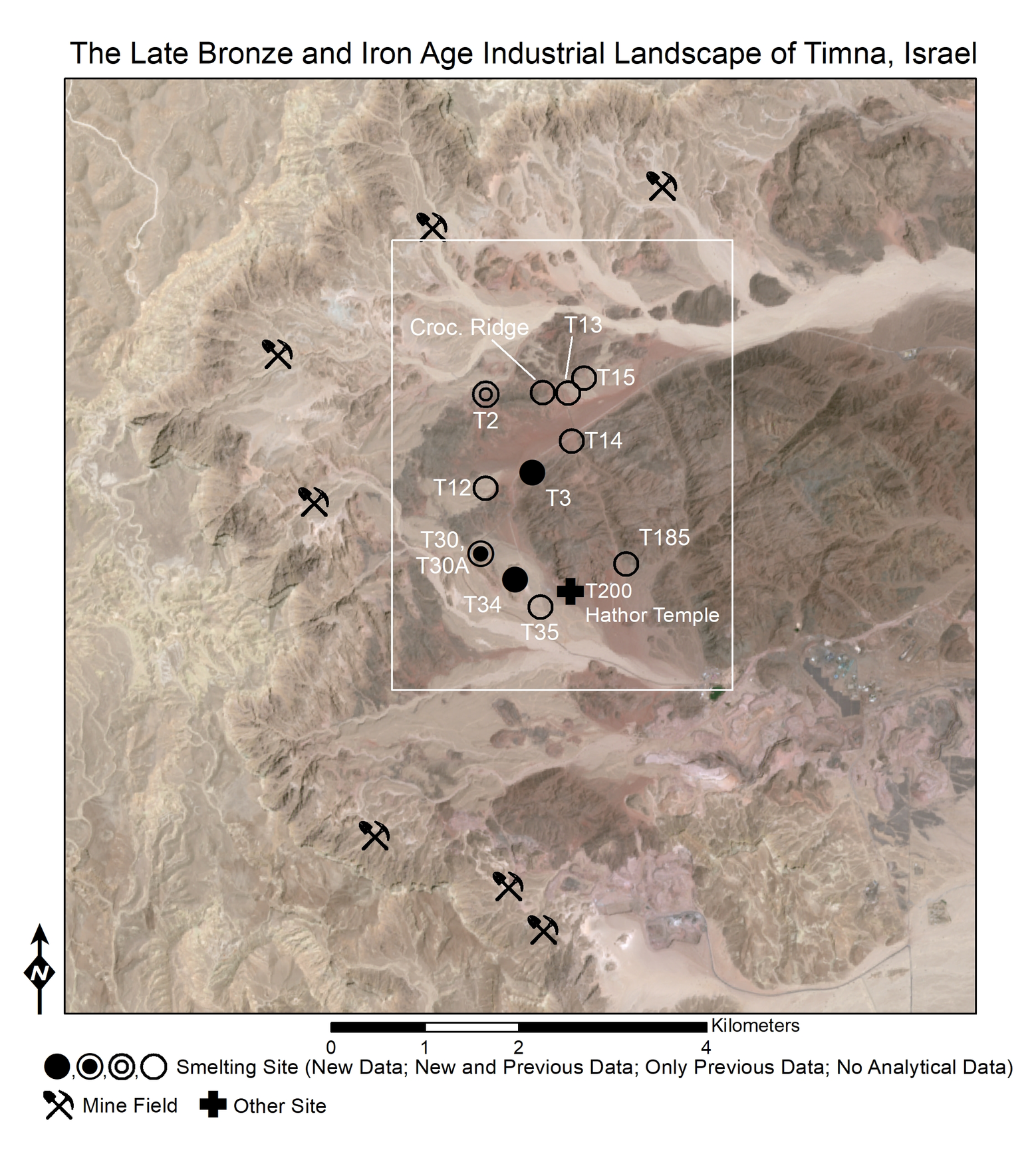

Supplement: S2 Fig — The white square represents the extent of the map provided in Fig 1. The site numbers are based on the work of the Arabah Expedition. Map produced using ArcGIS software by ESRI. Sentinel-2 (ESA) image courtesy of the U.S. Geological Survey (public domain). (TIF) [file pone.0221967.s002.tif]

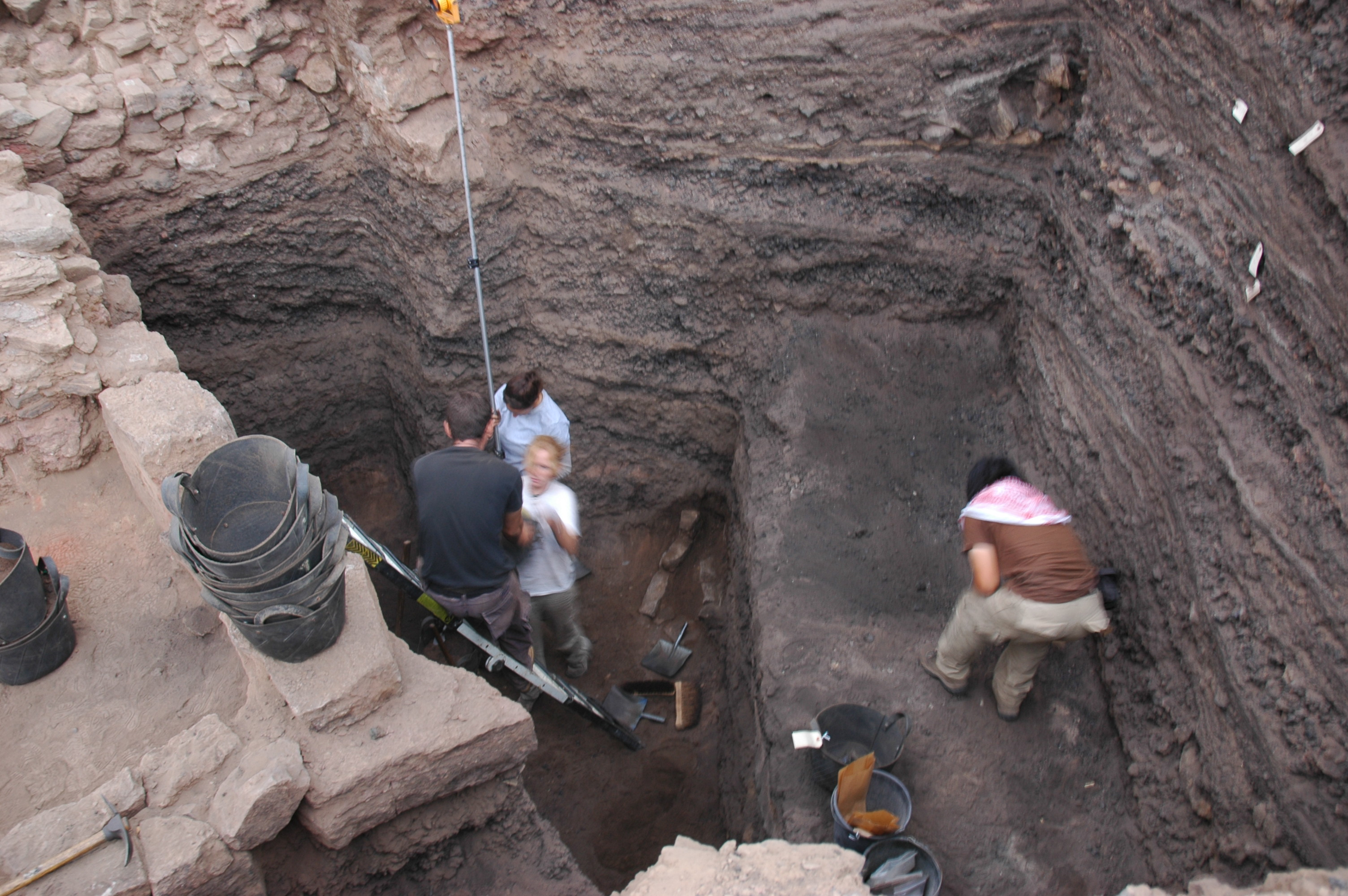

Supplement: S3 Fig — The rapidly-accumulating slag constitutes a quasi-continuous record of technological change (in this case, spanning 300 years) that can be tightly tied to chronology based on radiocarbon dating of charcoal (remains of fuel) and archaeomagnetic investigation of the slag material itself. The technological “leap” detected by the current study is represented here by the reorganization of the area during the second half of the 10th century BCE: the construction of the building on the left, and the leveling-up of the earlier slag mound represented as a ‘disruption’ in the section at the level of the building’s foundation. (TIF) [file pone.0221967.s003.tif]
